# National Hospital for the Paralysed and Epileptic

**Published:** 1901-04-13

**Authors:** G. H. Bower

**Affiliations:** 4 Bream's Buildings, Chancery Lane, E.C.


### Editor's Letter-Box.

NATIONAL HOSPITAL FOR THE PARALYSED AND EPILEPTIC.

To the Editor of *The Hospital*.

Sir,—The article appearing in your issue of March 30th and headed "An Ill-fated Hospital" has come under my notice.

It is not correct to say that after starting by saying that I would justify the Board I "not only failed to do so but threw up the sponge." This appears in your article on p. 448. On p. 462 of the same issue you give with substantial accuracy what I actually did say.

I intervened in the discussion for a definite purpose, viz. to accept Mr. Melvill Green's amendment after explaining that the resolutions proposed by the Board were designed to lead to a full inquiry into all matters including the important matter of the representation of the staff.

I submit that your report of my speech shows that I carried out my purpose. I made it plain that the Board had not in any way attempted to limit the inquiry, and I ended by accepting on behalf of the Board Mr. Melvill Green's amendment which practically carried out what the Board desired. This appears clearly from my speech.

I much regret to see the further suggestion in your article that the dispute has been throughout "a battle between the solicitor, Mr. Bower, acting through Mr. Rawlings, the secretary-director, and the medical staff backed by the medical profession," and you suggest that the Board have abrogated their functions and sacrificed their authority.

These suggestions have no foundation in fact. I have no desire, and I know that Mr. Burford Rawlings has no desire, to shirk proper responsibility, but it is unfair both to the Board and to us to suggest that the Board have been improperly or unduly subservient to our views.

You also refer to "Mr. Bower's policy of non-publication of the material points of the evidence." Here again my remarks are reported with substantial accuracy on p. 463 and do not support your remarks. Sir Henry Burdett proposed that everything said on both sides should be printed and circulated to the Governors. I, on the contrary, took the view that, although the *Report* must of course be circulated, it would be extremely unwise to decide beforehand that the whole evidence should be treated in the same way. What I said was "Leave this to the Committee," and I venture to suggest that my action goes to prove the sincerity of my desire that out of this dispute may issue peace.

One word as to the attitude and position of the Board.

The Board thought it right to try and agree with the medical staff a basis of inquiry. Their efforts having failed, they called the Governors together to decide the points as to which an agreement had not been arrived at, and they contemplated that when these points had been decided by the Governors the inquiry would proceed on the footing of the resolutions passed by the Board and the staff respectively, subject, of course, to the decision of the Governors on the disputed points. So that the inquiry be complete, searching, impartial, and authoritative, it matters not whether it is set on foot by means of an arrangement between the Board and the staff, or, as will now be the case, by means of independent action by selected Governors with the concurrence of both the immediate parties to the dispute.

As I said at the meeting, we all hope that the report on this inquiry will bring peace, and by no one is this hope more earnestly entertained than by myself.

I am, Sir, your obedient servant,

G. H. Bower.

4 Bream's Buildings, Chancery Lane, E.C.
April 3rd, 1901.

